# Genotype- and sex-specific changes in vital parameters during isoflurane anesthesia in a mouse model of Alzheimer’s disease

**DOI:** 10.3389/fmed.2024.1342752

**Published:** 2024-03-27

**Authors:** Sebastian Bratke, Sebastian Schmid, Bernhard Ulm, Bettina Jungwirth, Manfred Blobner, Laura Borgstedt

**Affiliations:** ^1^Department of Anesthesiology and Intensive Care Medicine, Faculty of Medicine, University of Ulm, Ulm, Germany; ^2^Department of Anesthesiology and Intensive Care Medicine, School of Medicine, Technical University of Munich, Munich, Germany

**Keywords:** Alzheimer’s disease, Tg2576, isoflurane, general anesthesia, vital parameters, mouse model, transgenic mice

## Abstract

**Background:**

The prevalence of neurodegenerative diseases is increasing as is life expectancy with Alzheimer’s disease accounting for two-thirds of dementia cases globally. Whether general anesthesia and surgery worsen cognitive decline is still a matter of debate and most likely depending on the interplay of various influencing factors. In order to account for this complexity, Alzheimer’s disease animal models have been developed. The Tg2576 model of Alzheimer’s disease is a well-established mouse model exhibiting amyloidopathy and age-dependent sex-specific differences in Alzheimer’s disease symptomology. Yet, data on anesthesia in this mouse model is scarce and a systematic comparison of vital parameters during anesthesia with wild-type animals is missing. In order to investigate the safety of general anesthesia and changes in vital parameters during general anesthesia in Tg2576 mice, we did a secondary analysis of vital parameters collected during general anesthesia in aged Tg2576 mice.

**Methods:**

After governmental approval (General Administration of the Free State of Bavaria, file number: 55.2-1-54-2532-149-11) 60 mice at 10-12 months of age were exposed to isoflurane (1.6 Vol%) for 120 min, data of 58 mice was analyzed. During general anesthesia, heart rate, respiratory rate, temperature, isoflurane concentration and fraction of inspired oxygen were monitored and collected. Data were analyzed using univariate and multivariate linear mixed regression models.

**Results:**

During general anesthesia, heart rate decreased in a sex-specific manner. Respiratory rate decreased and body temperature increased dependent on genotype. However, the changes were limited and all vital parameters stayed within physiological limits.

**Conclusion:**

Isoflurane anesthesia in the Tg2576 mouse model is safe and does not seem to influence experimental results by interacting with vital parameters. The present study provides information on appropriate anesthesia in order to advance research on anesthesia and AD and could contribute to improving laboratory animal welfare.

## 1 Introduction

According to the World Health Organization (WHO) 55 million people worldwide are currently living with dementia, with Alzheimer’s disease (AD) being the underlying cause in 60–80% of all cases. This number of AD patients is projected to increase to 139 million people by 2050 accounting for 1.6 trillion USD in healthcare costs by 2050 (1, 2).

With an increase in life expectancy as well as medical progress, patients with a preexisting cognitive impairment or even a diagnosis of AD might require surgery and thus either general or regional anesthesia. When it comes to general anesthesia (GA), it is still unclear whether general anesthetics aggravate a preexisting cognitive dysfunction or accelerate cognitive decline (3, 4). Investigating the effect of general anesthesia on AD pathology in humans is impeded by confounders such as surgical trauma, comorbidities, preoperative fasting and post-operative complications (5).

To account for these limitations and to advance research on AD pathology, diagnosis and therapy, various AD mouse models have been developed (6). Mice have a complex nervous system with notable homologies to humans in anatomy (7) and function in terms of learning, memory and behavior (8). There are numerous transgenic, knock-in, injection, and neuroinflammation based AD mouse models, representing amyloidopathy, tauopathy or both (6). In our study, the Tg2576 model was used. It overexpresses the human amyloid precursor gene with the KM670/671NL (so called “Swedish mutation”) under a viral hamster prion promotor and was first described by Hsiao et al. (9). These animals show first symptoms of cognitive decline at the age of 10 months, with sex specific differences at the age of 12 months, along with amyloid plaques and microglial activation in the neocortex and hippocampus (10, 11).

The monitoring of vital parameters is a standard procedure and ensures adequate and safe anesthesia by detecting and thus preventing hypotension, subsequent cerebral hypoperfusion or hypoxia which can lead to an unfavorable cognitive outcome (12). Although the Tg2576 mouse model has been used for many years and has often been subjected to anesthesia in this context, the authors are not aware of any publications regarding the associated changes in vital parameters (13, 14). In some cases, very limited monitoring was carried out (15, 16). Furthermore, despite the frequent use of isoflurane for general anesthesia in Tg2576 mice, literature on the effects of 120 min of general anesthesia on this mouse model is scarce (15, 17). To our knowledge, a direct comparison between the vital signs of anesthetized wild-type animals and Tg2576 has not yet been carried out systematically. In a previously published study, our group investigated the influence of isoflurane anesthesia on neurocognition, behavior and amyloidopathy in 10 months old Tg2576 mice with respect to sex. Typical symptoms of early stage AD with corresponding histopathological alterations were found, whereas relevant sex-specific differences or an influence of isoflurane on AD symptomology and pathology could not be detected (18). We retrospectively assessed vital parameters during general anesthesia in order to address whether isoflurane affects heart rate, respiratory rate and body temperature dependent on transgenic status or sex. Data were obtained during isoflurane anesthesia in Tg2576 and wild type mice randomized to intervention during the above-mentioned study. Concerning laboratory animal welfare, another aim of this publication was to evaluate the safety and physiological changes of Tg2576 under general isoflurane anesthesia (19).

## 2 Materials and Methods

This study was carried out in strict accordance with the recommendations of the Federation of European Laboratory Animal Science Associations (FELASA). The following experimental procedures on animals were approved by the Governmental Animal Care Committee (Regierung von Oberbayern, Maximilianstr. 39, 80538 Munich, Germany, Chair: Dr. B. Wirrer, Registration number: 55.2-1-54-2532-67-2016, July 28th, 2016). All efforts were made to minimize suffering. Animal welfare was assessed daily.

### 2.1 Mouse model

We used the B6; SJL-Tg (APPSWE) 2576Kha mouse model of AD, also referred to as Tg2576. With the approval of Taconic (Taconic Europe, Lille Skensved, Denmark), male Tg2576 mice were crossed with female C57B6/SJL mice (The Jackson Laboratory, Bar Harbor, ME, USA) in a separate breeding facility. The genotype was confirmed by PCR, using DNA from tail tissues (Charles River Laboratories, Sulzfeld, Germany). Mice homozygous for the rd1-mutation were excluded from the analysis as these mice are blind. At least 14 days prior to anesthesia, cognitive and behavioral testing, mice were transferred to a test facility for acclimatization. Mice were housed under standard laboratory conditions (specific pathogen free environment, 12 h light/12 h dark cycle, 22°C, 60% humidity and free access to water and standard mouse chow) (11, 18).

Mice were randomly assigned to the experimental groups regarding isoflurane anesthesia or sham procedure using a computer-generated randomization list (18).

### 2.2 General anesthesia

For induction of general anesthesia mice were placed in an acrylic glass chamber that had been pre-flushed with 4.5 Vol% isoflurane (Isofluran Baxter vet, Deerfield, IL, USA; Vaporizer: Draeger, Lübeck, Germany) and 50% oxygen. After loss of postural reflexes, mice were placed in sternal recumbency on a warming pad. General anesthesia was maintained for 120 min with 1.6 Vol% isoflurane (MAC 1.0) and a fraction of inspired oxygen of 50% (FiO2 0.5) administered via a nose chamber. Mice breathed spontaneously with an applied positive end-expiratory pressure of 5 mbar. Anesthetic depth was monitored every 15 min using the tail clamp test (20). Eye lubricant (Bepanthen^®^, Bayer Vital GmbH, 51368 Leverkusen, Germany) was applied and animals were individually covered with compresses (Vliwasoft^®^, Lohmann & Rauscher GmbH & Co. KG, 56567 Neuwied, Germany).

After 120 min mice were placed in the acrylic glass chamber again with 50% oxygen now without isoflurane until full recovery from anesthesia. Afterward the animals were weighed and placed in single cages. During general anesthesia respiratory rate, heart rate (both via subcutaneous electrocardiogram), gas concentrations, and rectal temperature were measured (Datex Ohmeda S/5 Anesthesia Monitor F-CM1-05 with MNESTPR Modul, Datex-Ohmeda GmbH, Duisburg, Germany).

### 2.3 Statistical analysis

All analyses were conducted with RStudio 2023.09.1 (RStudio, Boston, MA, USA) running R version 4.3.1 (R Foundation for Statistical Computing, Vienna, Austria).

Categorical values are presented as absolute and relative numbers, continuous variables with median and interquartile range (IQR). For group comparisons Mann-Whitey *U*-tests and Kruskal-Wallis tests with Bonferroni’s corrected *post-hoc* tests were used. To assess changes in vital parameters during the time of anesthesia in combination with the possibly influencing factors sex and genotype, linear mixed regression models were calculated. An alpha of 5% was seen as significant.

## 3 Results

A total of 60 mice (median weight 28.1 g) underwent general anesthesia for 120 min (Table 1). Incomplete recordings in two mice due to a technical failure at the start of the experiments led to the exclusion of these animals from further analyses concerning general anesthesia. Therefore 58 animals in total with a median age of 10.5 months were analyzed, of which 27 (46.6%) were male. Genotype was equally distributed (Table 1).

**TABLE 1:** Depiction of experimental groups.

	*n* = 58
**Sex, *n* (%)**	
Male	27 (46.6)
Female	31 (53.4)
**Genotype, *n* (%)**	
Tg2576	29 (50.0)
WT	29 (50.0)
**Combination of sex and genotype, *n* (%)**	
m_Tg2576	14 (24.1)
m_WT	13 (22.4)
f_Tg2576	15 (25.9)
f_WT	16 (27.6)
**RD1 status, *n* (%)**	
Heterozygous	31 (53.4)
WT	27 (46.6)
**Age (months), Median (IQR)**	10.5 (10–12)

### 3.1 Heart rate

**FIGURE 1:**
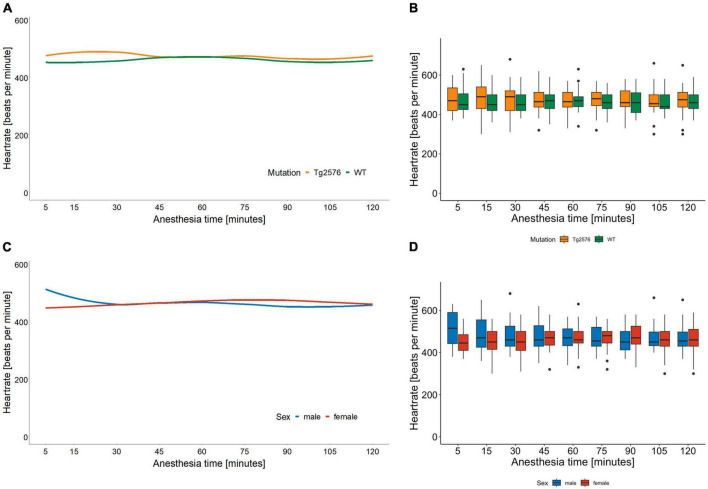
Heart rate in beats per minute and anesthesia time in minutes sorted by genotype **(A)** and corresponding boxplots over a 15 min interval **(B)**; median (horizontal lines), interquartile range (box) and range (whiskers), dots represent outliers at least 1.5 times of the interquartile range (IQR). The heart rate of Tg2576 mice and WT mice did not show significant differences. Heart rate in beats per minute and anesthesia time in minutes sorted by sex **(C)** and corresponding boxplots over a 15 min interval **(D)**; median (horizontal lines), interquartile range (box) and range (whiskers), dots represent outliers at least 1.5 times of the interquartile range (IQR). The heart rate of female animals was lower at the beginning of anesthesia and increased over the course of anesthesia. In male animals, the heart rate was higher at the beginning and decreased over time, with a tendency toward approximation in both sexes.

The median heart rate was 466 (434–499) beats per minute (bpm) in all animals during general anesthesia, without significant sex- or genotype-specific differences in univariate analyses (Table 2). Anesthesia time [min], had a significant influence on heart rate [−0.26 (−0.38 to −0.13); *p* < 0.001, Table 3] as heart rate decreased over time in univariate analyses (Figures 1A, B).

**TABLE 2:** Univariate analysis of vital parameters stratified by sex and genotype.

	Overall, *N* = 58					
Heart rate, median (IQR)	466 (434–499)					
Respiratory rate, median (IQR)	104 (93–115)					
Body temperature, median (IQR)	36.8 (36.5–37.6)					
	**Sex**					
	**Male, *N* = 27**	**Female, *N* = 31**	***p*-value**			
Heart rate, median (IQR)	454 (432–507)	468 (438–492)	0.97			
Respiratory rate, median (IQR)	103 (92–114)	106 (93–117)	0.58			
Body temperature, median (IQR)	36.8 (36.7–37.8)	36.8 (36.4–37.6)	0.39			
	**Genotype**					
	**Tg2576, *N* = 29**	**WT, *N* = 29**	***p*-value**			
Heart rate, median (IQR)	476 (442–500)	454 (430–493)	0.48			
Respiratory rate, median (IQR)	96 (89–109)	111 (100–121)	**0.005**			
Body temperature, median (IQR)	36.7 (36.3–37.2)	37.2 (36.8–38.1)	**0.022**			
	**Combination of sex and genotype**					
	**Male Tg2576, *N* = 14**	**Male WT, *N* = 13**	**Female Tg2576, *N* = 15**	**Female WT, *N* = 16**	***p*-value**	
Heart rate, median (IQR)	476 (445–503)	436 (421–510)	476 (438–492)	466 (445–492)	0.69	
Respiratory rate, median (IQR)	94 (90–107)	112 (103–120)	96 (87–112)	111 (100–121)	**0.039**	
Body temperature, median (IQR)	36.8 (36.5–37.2)	37.0 (36.8–38.1)	36.4 (36.3–37.2)	37.2 (36.7–37.8)	0.093	
	***Post hoc***					
	**Male Tg2576 male WT**	**Male Tg2576 female Tg2576**	**Male Tg2576 female WT**	**Male WT female Tg2576**	**Male WT female WT**	**Female Tg2576 female WT**
Heart rate, median (IQR)						
Respiratory rate, median (IQR)	<0.001	0.41	<0.001	<0.001	1	<0.001

Body temperature, median (IQR). Significance level was set at *p* < 0.05.

**TABLE 3:** Multivariate linear mixed regression models with variables time, sex, genotype and combination of sex and genotype for heart rate, respiratory rate and body temperature.

	Heart rate		Respiratory rate		Body temperature	
	**Beta (95% CI)**	***p*-value**	**Beta (95% CI)**	***p*-value**	**Beta (95% CI)**	***p*-value**
**Sex**						
Anesthesia time	−0.26 (−0.38 to −0.13)	**<0.001**	−0.14 (−0.18 to −0.10)	**<0.001**	0.01 (0.01 to 0.01)	**<0.001**
Sex		**0.020**		0.97		0.25
Male	–		–		–	
Female	−35 (−65 to −5.6)		0.21 (−9.4 to 9.9)		−0.29 (−0.79 to 0.21)	
Anesthesia time × Sex		**<0.001**		0.62		0.074
Anesthesia time × Female	0.42 (0.25 to 0.59)		0.01 (−0.04 to 0.07)		0.00 (0.00 to 0.00)	
**Genotype**						
Anesthesia time	−0.06 (−0.18 to 0.06)	0.31	−0.14 (−0.18 to −0.10)	**<0.001**	0.01 (0.01 to 0.01)	**<0.001**
Genotype		0.47		**0.007**		0.10
Tg2576	–		–		–	
WT	−11 (−41 to 19)		12 (3.4 to 21)		0.41 (−0.08 to 0.89)	
Anesthesia time × Genotype		0.37		0.74		0.99
Anesthesia time × WT	0.08 (−0.09 to 0.25)		0.01 (−0.05 to 0.07)		0.00 (0.00 to 0.00)	
**Combination of sex and genotype**						
Anesthesia time	−0.40 (−0.57 to −0.22)	**<0.001**	−0.18 (−0.24 to −0.12)	**<0.001**	0.01 (0.01 to 0.01)	**<0.001**
Combination of sex and genotype		**0.025**		0.059		0.23
Male Tg2576	–		–		–	
Male WT	−42 (−86 to 2.1)	0.063	8.5 (−4.9 to 22)		0.27 (−0.45 to 1.0)	
Female Tg2576	−63 (−106 to −21)	**0.003**	−3.6 (−17 to 9.3)		−0.43 (−1.1 to 0.26)	
Female WT	−48 (−90 to −6.2)	**0.025**	12 (−1.1 to 24)		0.10 (−0.59 to 0.78)	
Anesthesia time × Combination of sex and genotype		**<0.001**		0.14		0.33
Anesthesia time × Male WT	0.28 (0.03 to 0.53)	**0.026**	0.08 (0.00 to 0.17)		0.00 (0.00 to 0.00)	
Anesthesia time × Female Tg2576	0.61 (0.37 to 0.84)	**<0.001**	0.08 (0.00 to 0.16)		0.00 (0.00 to 0.01)	
Anesthesia time × Female WT	0.52 (0.28 to 0.76)	**<0.001**	0.03 (−0.05 to 0.11)		0.00 (0.00 to 0.01)	

Significance level was set at *p* < 0.05.

Overall, females had a significantly lower heart rate compared to males [−35 (−65 to −5.6) bpm; *p* = 0.020, Table 3] without a significant influence of genotype [−11 (−41 to 19); *p* = 0.47, Table 3 and Figures 1C, D].

There was a significant interaction between anesthesia time and sex with a positive value for females over time [0.42 (0.25 to 0.59); *p* < 0.001, Table 3]. The heart rate in female mice was lower at the beginning and increased over the course of anesthesia (Figures 1C, D).

The combination of sex and genotype showed a significantly negative influence of female sex in both Tg2576 [−63 (−106 to −21); *p* = 0.003] and WT [−48 (−90 to −6.2); *p* = 0.025] on heart rates (Table 3) compared to male Tg2576 as the heart rates of male Tg2576 remained above those of the other experimental groups throughout general anesthesia.

Anesthesia time had a significantly positive effect on the combination of sex and genotype in male WT [0.28 (0.03 to 0.53); *p* = 0.026], female Tg2576 [0.61 (0.37 to 0.84); *p* < 0.001] and female WT [0.52 (0.28 to 0.76); *p* < 0.001, Table 3].

Female Tg2576 had a significantly lower heart rate [−63 (−108 to −19); *p* = 0.005, Table 4] than male Tg2576.

**TABLE 4:** Multivariate linear mixed regression model with variables time, sex, genotype and further stratification by experimental group for heart rate, respiratory rate and body temperature.

			Heart rate		Respiratory rate		Body temperature	
			**Beta** **(95% CI)**	***p*-value**	**Beta** **(95% CI)**	***p*-value**	**Beta** **(95% CI)**	***p*-value**
Genotype	Tg2576	Anesthesia time	−0.40 (−0.59 to −0.21)	**<0.001**	−0.18 (−0.25 to −0.12)	**<0.001**	0.01 (0.00 to 0.01)	**<0.001**
		Sex		**0.005**		0.59		0.23
		Male	–		–		–	
		Female	−63 (−108 to −19)		−3.7 (−17 to 9.8)		−0.43 (−1.1 to 0.27)	
		Anesthesia time × Sex		**<0.001**		0.051		0.13
		Anesthesia time × Female	0.61 (0.35 to 0.86)		0.08 (0.00 to 0.16)		0.00 (0.00 to 0.01)	
	WT	Anesthesia time	−0.11 (−0.27 to 0.05)	0.16	−0.10 (−0.16 to −0.04)	**<0.001**	0.01 (0.01 to 0.01)	**<0.001**
		Sex		0.76		0.62		0.61
		Male	–		–		–	
		Female	−6.2 (−46 to 34)		3.2 (−9.3 to 16)		−0.18 (−0.87 to 0.51)	
		Anesthesia time × Sex		**0.032**		0.21		0.31
		Anesthesia time × Female	0.24 (0.02 to 0.45)		−0.05 (−0.13 to 0.03)		0.00 (0.00 to 0.00)	
Sex	Male	Anesthesia time	−0.40 (−0.58 to −0.22)	**<0.001**	−0.18 (−0.24 to −0.13)	**<0.001**	0.01 (0.01 to 0.01)	**<0.001**
		Genotype		0.076		0.11		0.43
		Tg2576	–		–		–	
		WT	−42 (−88 to 4.3)		8.6 (−1.8 to 19)		0.27 (−0.41 to 0.96)	
		Anesthesia time × Genotype		**0.027**		**0.030**		0.66
		Anesthesia time × WT	0.29 (0.03 to 0.54)		0.08 (0.01 to 0.16)		0.00 (0.00 to 0.00)	
	Female	Anesthesia time	0.21 (0.05 to 0.36)	**0.009**	−0.10 (−0.16 to −0.04)	**<0.001**	0.01 (0.01 to 0.01)	**<0.001**
		Genotype		0.44		**0.037**		0.14
		Tg2576	–		–		–	
		WT	15 (−24 to 54)		15 (0.93 to 30)		0.53 (−0.16 to 1.2)	
		Anesthesia time × Genotype		0.45		0.25		0.77
		Anesthesia time × WT	−0.09 (−0.31 to 0.14)		−0.05 (−0.13 to 0.03)		0.00 (0.00 to 0.00)	

Significance level was set at *p* < 0.05.

The combination of anesthesia time and female sex showed a significantly positive influence on heart rate in Tg2576 [0.61 (0.35 to 0.86); *p* < 0.001, Table 4] and WT [0.24 (0.02 to 0.45); *p* = 0.032, Table 4].

In male mice, WT genotype had a significantly positive influence on heart rate [0.29 (0.03 to 0.54); *p* = 0.027] when assessing the combination of anesthesia time and genotype (Table 4).

### 3.2 Respiratory rate

The median respiratory rate was 104 (93–115) breaths per minute in all animals during general anesthesia (Table 2).

Univariate comparisons showed a significant difference between genotype [Tg2576 96 (89–109) and WT 111 (100–121); *p* = 0.005, Table 2] and between the combination of sex and genotype [male Tg2576 94 (90–107), male WT 112 (103–120), female Tg2576 96 (87–112), female WT 111 (100–121); *p* = 0.039, Table 2] in median respiratory rates.

**FIGURE 2:**
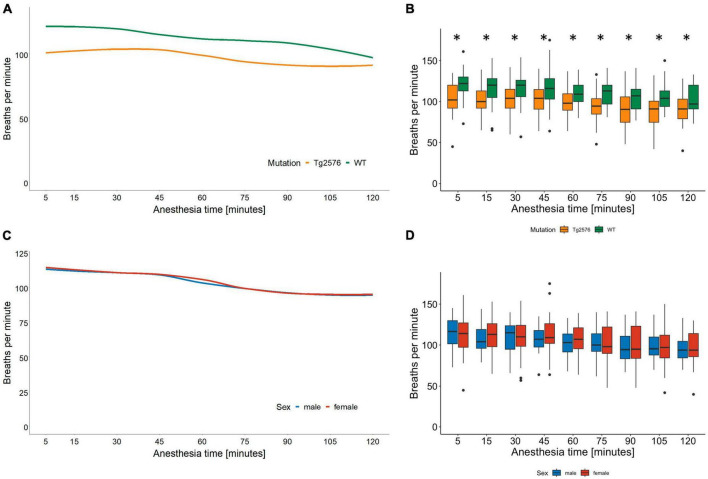
Respiratory rate in breaths per minute and anesthesia time in minutes sorted by genotype **(A)** and corresponding boxplots over a 15 min interval **(B)**; median (horizontal lines), interquartile range (box) and range (whiskers), dots represent outliers at least 1.5 times of the interquartile range (IQR), *p = 0.007. The respiratory rate in Tg2576 mice was significantly lower compared to WT mice. Respiratory rate in breaths per minute and anesthesia time in minutes sorted by sex **(C)** and corresponding boxplots over a 15 min interval **(D)**; median (horizontal lines), interquartile range (box) and range (whiskers), dots represent outliers at least 1.5 times of the interquartile range (IQR). The respiratory rate decreased in all animals over the course of isoflurane anesthesia, without a statistically significant difference between sex.

Overall, WT animals had a significantly higher respiratory rate throughout general anesthesia [12 (3.4 to 21); *p* = 0.007, Table 3 and Figures 2A, B].

In male animals, WT genotype significantly positively influenced respiratory rate [0.08 (0.01 to 0.16); *p* = 0.030, Table 4].

### 3.3 Body temperature

The median body temperature in all animals was 36.8 (36.5–37.6)°C (Table 2) with a significant difference in genotype [Tg2576 36.7 (36.3–37.2)°C, WT 37.2 (36.8–38.1)°C; *p* = 0.022] in univariate comparisons (Table 2).

**FIGURE 3:**
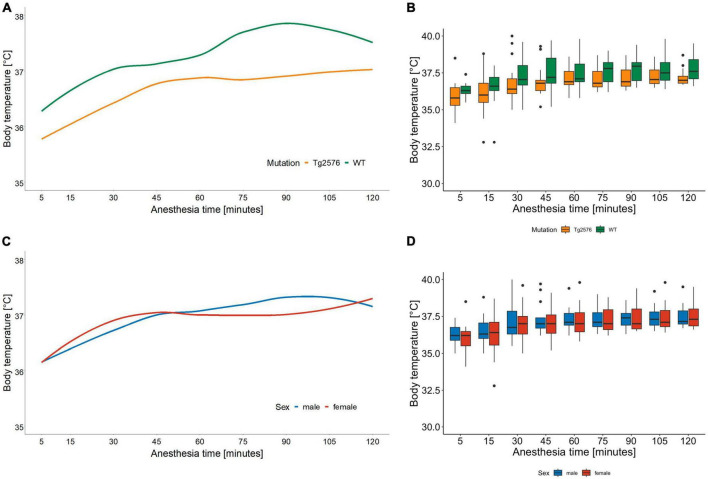
Body temperature in °C and anesthesia time in minutes sorted by genotype **(A)** and corresponding boxplots over a 15 min interval **(B)**; median (horizontal lines), interquartile range (box) and range (whiskers), dots represent outliers at least 1.5 times of the interquartile range (IQR). Body temperature of Tg2576 and WT mice differ over the course of anesthesia. Throughout anesthesia body temperature increased in all animals, without a statistically significant difference between genotype. Body temperature in °C and anesthesia time in minutes sorted by sex **(C)** and corresponding boxplots over a 15 min interval **(D)**; median (horizontal lines), interquartile range (box) and range (whiskers), dots represent outliers at least 1.5 times of the interquartile range (IQR). Body temperature increased in all animals over the course of anesthesia, without a statistically significant difference between sex.

During anesthesia body temperature increased over time [0.001 (0.01 to 0.01); *p* < 0.001] without significant differences in regression models (Tables 3, 4 and Figures 3A–D).

Over the time of anesthesia, all vital parameters changed significantly, except for the model of heart rate and genotype [−0.06 (−0.18 to 0.06), *p* = 0.31, Table 3].

## 4 Discussion

This study is a secondary analysis of data collected during general anesthesia in a mouse model of AD. Heart rate, respiratory rate and body temperature of both male and female 10–12 months old Tg2576 and wild type mice under isoflurane anesthesia were analyzed. We found time dependent as well as sex- and genotype-specific differences in vital parameters. After general anesthesia, mice underwent neurocognitive testing for 8 consecutive days. Results from the post-anesthesia part of the study have already been published (18). Data on anesthesia in Tg2576 mice over a period of 120 min are not very frequently encountered in the literature, as general anesthesia in animal experiments is often maintained for a shorter time period and published data on vital parameters is limited (13, 15, 17, 21). In view of this, these results can be considered a valuable contribution to the field of experimental AD research and provide a unique insight into the physiology of Tg2576 during general anesthesia.

Since its first description in 1996 (9), the Tg2576 animal model has been widely used in AD research to elucidate AD pathophysiology (10, 22–25), symptomology (26–28) and possible therapeutic strategies for this to date incurable neurodegenerative disease (29–33). Also, studies on the effect of general anesthesia on AD pathology have been done using this AD mouse model (17, 18, 21). Tg2576 animals have been very well described (34, 35) and are known to exhibit sex-specific differences similar to human AD pathology (11). In order to translate experimental evidence into the human organism, valid animal models are warranted. Although the Tg2576 animal model has already been well characterized in different aspects of AD research, literature on vital parameters during general anesthesia is limited. We therefore undertook a secondary analysis of heart rate, respiratory rate and body temperature in the Tg2576 mouse model taken during general anesthesia.

We found that the median heart rate did not differ significantly between animals in univariate analyses. However, in multivariate analyses female mice had a lower heart rate which increased over the course of anesthesia while the heart rate in male mice decreased, with a tendency toward approximation (Figures 1C, D). Heart rates in male Tg2576 and female mice approximated toward the end of general anesthesia (Figures 1A, B). This finding is in line with other studies on general anesthesia in laboratory animals, where heart rates in female (36) and animals of both sex (37, 38) increased over the course of isoflurane anesthesia. In (37) sex was not a significant factor for heart rate in C57BL/6J mice whereas our data showed a significant difference between male and female Tg2576 and WT mice. There is emergent evidence of a heart-brain-interaction in AD in terms of increased heart rate variability in males associated with better cognitive resilience (39) and susceptibility to AD pathology (40), so a sex-dependent effect on the heart rate based on the respective genotype in this AD mouse model cannot be excluded. Also, isoflurane is known to induce hypotension (41) and an increase in heart rate could be a compensatory mechanism. We did not measure blood pressure during anesthesia, which on the one hand can be viewed as a limitation of our study. On the other hand, it must be noted that the American Heart Association recommended avoiding anesthesia during blood pressure measurement due to the effect of anesthetics on cardiovascular function (42). We aimed to avoid invasive blood pressure measurement techniques in order to minimize distress for the animals and decided against a tail-cuff method since mice have complex thermoregulating processes involving fluctuating vasomotor tone of the tail, especially under general anesthesia (43). For the same reason we refrained from taking blood samples.

The respiratory rate decreased in all animals during isoflurane anesthesia (Figures 2C, D). Tg2576 mice had a lower respiratory rate than WT littermates without significant sex-specific differences (Figures 2A, B). Again, these observations reflect the current literature on isoflurane anesthesia in mice (36–38) in terms of a decrease in absolute numbers during anesthesia. It has to be noted that respiratory rates in our study ranged between 94 and 111 breaths per minute, whereas other publications stated respiratory rates of <80 per minute (37), <60 per minute (36), and <100 per minute (38). These differences in respiratory rates might be due to different isoflurane concentrations and subsequently differing anesthetic depths. In our study, 4.5 Vol% of isoflurane was administered for induction of general anesthesia, followed by 1.6 Vol% for anesthesia maintenance. Isoflurane concentration was reduced to 1.6 Vol% immediately after loss of righting reflex. This concentration was found to be sufficient by tail clamp test assessment while other authors used isoflurane concentrations of 1.5–2.1% (37), 2.8% (36), and 2% (38). Several aspects have to be considered when putting these data into perspective. The cited studies used C57BL/6J mice (36, 37) or ddY mice (38) at a significantly younger age [6 weeks (36), 8–20 weeks (37), and 7 weeks (38)] and applied isoflurane anesthesia for a shorter time [40 min (38), 50 min (36) and 60 min (37)] compared to the 120 min of anesthesia in our experiments. It is known that genetic variability influences susceptibility to isoflurane in mouse strains (44) and humans (45) and that anesthetic requirement decreases with age (46), which might account for the differences observed in our data. SpO2 was not measured in our study which is a limitation. However, other animal studies found SpO_2_ to be stable even with lower respiratory rates (37, 38), although it has to be noted that fractions of inspired oxygen as high as 100% were used.

Throughout the course of anesthesia body temperature increased in all animals while actively warmed using a warming pad (Figures 3C, D). Hypothermia is a known complication in general anesthesia with numerous deleterious effects on both human (47) and laboratory animal (48) physiology. Body temperature correlated positively with heart rate and respiratory rate in female C57BL/6J mice (48) during 30 min of isoflurane anesthesia which is again an anesthesia time shorter than in our study. Although animals were handled identically, we found that body temperature differed significantly between genotypes in univariate but not in multivariate analyses. Tg2576 had a median body temperature of 36.7°C compared to 37.2°C in WT (Figures 3A, B). This is reflective of AD pathology in humans where old age, disruption in thermoregulation and neurodegeneration seem to be interconnected (49).

## 5 Conclusion

In this secondary analysis of vital parameters collected during general anesthesia of Tg2576 mice and WT littermates we found marginal, yet significant differences in heart rate, respiratory rate and body temperature. Despite these differences, all vital parameters remained within physiological limits. Our findings indicate that isoflurane anesthesia in this AD mouse model is safe and does not seem to influence experimental results by interacting with vital parameters. The present study provides information on appropriate anesthesia in order to advance research on anesthesia and AD and could contribute to improving laboratory animal welfare.

## Data availability statement

The raw data supporting the conclusions of this article will be made available by the authors, without undue reservation.

## Ethics statement

The animal study was approved by the Regierung von Oberbayern, Maximilianstr. 39, 80538 Munich, Germany, Chair: Dr. B. Wirrer, Registration number: 55.2-1-54-2532-67-2016, July 28th, 2016. The study was conducted in accordance with the local legislation and institutional requirements.

## Author contributions

SB: Formal analysis, Investigation, Validation, Writing – review and editing. SS: Conceptualization, Methodology, Supervision, Writing – review and editing. BU: Formal analysis, Methodology, Validation, Writing – review and editing, BJ: Conceptualization, Supervision, Writing – review and editing. MB: Methodology, Resources, Supervision, Writing – review and editing. LB: Conceptualization, Data curation, Formal analysis, Investigation, Methodology, Validation, Writing – original draft.
